# Pharmacokinetics of Dexmedetomidine in an In Vitro Circuit Model and In Vivo Extracorporeal Membrane Oxygenation Rat Model

**DOI:** 10.7759/cureus.82165

**Published:** 2025-04-13

**Authors:** Yuhki Sato, Erina Mothoishi, Rina Kakuba, Yuka Nagatsuka, Takuya Abe, Yutaka Fujii

**Affiliations:** 1 Department of Pharmaceutical Sciences, Faculty of Pharmacy and Pharmaceutical Sciences, Fukuyama University, Fukuyama, JPN; 2 Department of Clinical Engineering and Medical Technology, Niigata University of Health and Welfare, Niigata, JPN

**Keywords:** dexmedetomidine, extracorporeal membrane oxygenation, noncompartmental analysis, nonlinear mixed-effects modeling, pharmacokinetics

## Abstract

Background and objectives: Extracorporeal membrane oxygenation (ECMO) causes variability in the pharmacokinetics (PK) of drugs. Fluctuations in the PK profile of dexmedetomidine (DEX) might significantly reduce the likelihood of obtaining a therapeutic effect. The study aimed to clarify the effect of ECMO on the PK of DEX.

Methods: *In vitro* circuit and* in vivo* rat models were developed, both of which involved a bolus injection of DEX and concentration monitoring. In the *in vivo* model experiment, rats that underwent ECMO were compared with a control group. PK analysis of *in vivo* data (noncompartmental analysis and nonlinear mixed-effects modeling) was carried out to determine the effect of ECMO.

Results: In the *in vitro* model experiment, the expected recovery (%) of DEX decreased after injection into the circuits. *In vivo* PK analysis showed that the ECMO significantly influenced the clearance of DEX, which was 75% lower in the presence of ECMO than in the absence of ECMO.

Conclusion: These results suggest that ECMO may affect the efficacy or increase the risk of side effects associated with DEX*.*

## Introduction

Critically ill patients can undergo pathophysiological changes, including septic shock, multiple organ dysfunction, and acute respiratory distress syndrome. Extracorporeal membrane oxygenation (ECMO) is a form of extracorporeal life support that provides prolonged cardiac and respiratory support to patients with hearts and lungs with reduced capacity to provide adequate oxygen, gas exchange, or blood supply to sustain life [1]. ECMO is also used to support patients with acute viral pneumonia associated with coronavirus disease 2019 when artificial ventilation alone is insufficient to sustain blood oxygenation [2].

Dexmedetomidine (DEX) is an alpha-2 adrenergic receptor agonist that works faster, with a rapid onset and offset of sedation in patients [3]. DEX can induce a sedative state similar to natural sleep so that patients under DEX are easily woken [4]. DEX is widely used for anesthesia under ECMO. DEX is metabolized into inert metabolites primarily through hydroxylation and glucuronic acid conjugation [5]. The sedative effect of DEX is dependent on its plasma concentration. Further, variations in patient characteristics and pharmacokinetics (PK) during ECMO might contribute to inconsistent clinical responses to DEX [6]. For critically ill patients, the effective blood concentration of DEX is 0.2-0.6 ng/mL; this ensures treatment efficacy regardless of intra-individual PK variability [3]. Pathophysiological changes appear to be most prevalent in critically ill patients, including those affected by septic shock, multiple organ dysfunction, and acute respiratory distress syndrome (ARDS). Adsorption by ECMO circuits is strongly affected by lipophilic and protein-binding characteristics [7-9]. The adsorption of drugs by ECMO circuits changes PK in patients and may also impact the pharmacological efficacy or side effects of drugs [10,11]. DEX and albumin associated with protein binding are adsorbed in the oxygenator and tube in the extracorporeal membrane oxygenation circuit [9,12-14]. At present, few data are available concerning the effect of ECMO on the PK of DEX.

The use of sedatives in critically ill patients is usually complicated due to the degree to which PK parameters are subject to inter-individual variability. One factor of inter-individual variability is ECMO, which induces considerable fluctuation in the concentrations of sedatives at the same dosage levels [15]. However, fluctuations in the PK profile of DEX might significantly reduce the likelihood of obtaining a therapeutic effect.

This study aimed to clarify the effect of ECMO on the PK of DEX. First, an *in vitro* circuit model was used to characterize the expected recovery (%) of DEX according to the drug concentration and the amount of elapsed time of contact with the ECMO circuit. Subsequently, complementary *in vivo* studies were performed using rat models with PK analysis (noncompartmental analysis and nonlinear mixed-effects modeling).

This article was previously posted to Preprints.org on September 11, 2024.

## Materials and methods

*In vitro *circuit experiment

In a closed *in vitro* ECMO circuit, a reservoir bottle (100 mL) and a centrifugal pump connected by a heparin-coated circuit tube without an oxygenator were prepared. A total of 15 mL of rat plasma was poured into the circuit as a packing liquid, and the circuit was set at the flow rate of 20 mL/minute. The pH and temperature in the circuit were maintained at 7.4 and 37 °C, respectively. At time = 0, DEX (10 µg) was injected as a bolus into the circuit; 0.5 mL of solution was collected at nine time points: two minutes to 24 hours after the initiation. After collection, the samples were pipetted into cryovials, which were immediately stored at −80 °C. The drug concentration was measured by liquid chromatography with mass spectrometry (LC-MS/MS).

Ethical approval

All experiments were approved by the Animal Care and Use Committee of Niigata University of Health and Welfare (Ethical Code 22003) and conducted in accordance with the guidelines of the National Institute of Health for Laboratory Animal Welfare. This study is reported in accordance with ARRIVE (Animal Research: Reporting of In Vivo Experiments) guidelines.

Anesthesia, surgical preparation, and ECMO

Adult male Sprague-Dawley rats (male, 14-16 weeks old, 400-450 g) were used for all experiments. The animals were obtained from CLEA Japan, Inc. (Tokyo, Japan). The experimental procedures were conducted as described by Fujii et al. [16]. The rats were anesthetized via inhalation of 5.0% isoflurane mixed with oxygen-enriched air delivered via a vaporizer. They were placed in the supine position, and a rectal temperature probe was inserted.The rats were orotracheally intubated using a 14-G cannula (Terumo Corp., Tokyo, Japan), and mechanical ventilation was initiated with a respirator for small animals (Model 683; Harvard Apparatus Ltd., Holliston, MA, USA). The tidal volume was 10 mL/kg, and the respiratory rate was 70 breaths/minute. Anesthesia was maintained with 2.0% isoflurane (without neuromuscular blocking agents), and the rectal temperature was maintained at physiological levels above 35.5 °C throughout the experiment. The right femoral artery was cannulated with polyethylene tubing (SP45, Natsume Seisakusho Co. Ltd., Tokyo, Japan) for monitoring systemic arterial blood pressure (BP) using Power Lab (ML880, AD Instruments, Bella Vista, NSW, Australia). A polyethylene tube (SP55, Natsume Seisakusho Co. Ltd.) was used to cannulate the left common carotid artery as the outflow cannula for the ECMO system. Heparin (500 IU/kg) was administered via an outflow cannula. The right external jugular vein was cannulated using a 16-G cannula (EB 16G 4HCLs×1･1/2"PP, Togomedkit Co. Ltd., Tokyo, Japan) to facilitate venous uptake. The ECMO system consisted of a polyvinyl chloride circuit (Senko Medical Co., Ltd., Tokyo, Japan), a small animal membrane oxygenator (membrane area 0.033 m^2^, polypropylene, Senko Medical Co., Ltd., Tokyo, Japan), and a roller pump (REGLO Digital ISM831, ISMATEC, Wertheim, Germany) primed with 7 mL of saline and 1 mL (1000 IU) of heparin.

Experimental design

**Figure 1:**
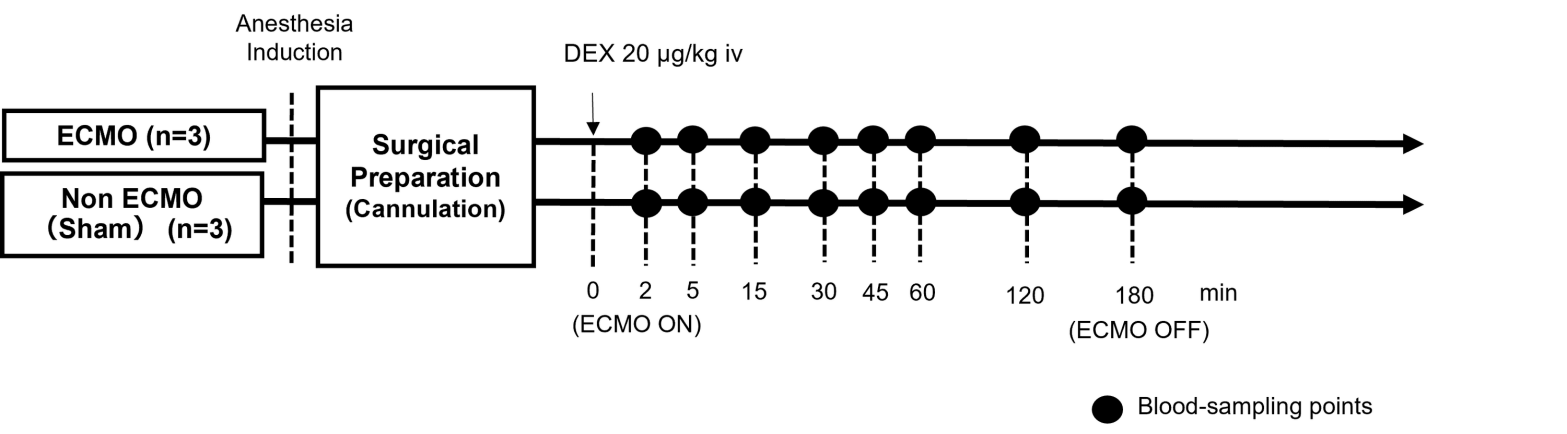
Schematic diagram of the experimental design. Six rats were divided into two groups: a DEX-treated ECMO group (ECMO rats, n = 3) and a DEX-treated non-ECMO group (sham rats, n = 3) in which the rats underwent surgical preparation without ECMO. Blood samples were collected at eight defined time points: two minutes after treatment with DEX (20 μg/kg body weight) before ECMO initiation and at 5-180 minutes after the initiation of ECMO. DEX: dexmedetomidine; ECMO: extracorporeal membrane oxygenation Image credits: Yuhki Sato

Six rats were divided into two groups: a DEX-treated ECMO group (ECMO rats, n = 3) and a DEX-treated non-ECMO group (sham rats, n = 3) in which the rats underwent surgical preparation without ECMO. The ECMO pump flow rate was targeted at 60-70 mL/kg/minute. The arterial pressures of carbon dioxide (PaCO_2_) and oxygen (PaO_2_) were maintained at 35-45 mmHg and 250-350 mmHg, respectively. Blood samples were collected at eight defined time points: two minutes after treatment with DEX (20 μg/kg body weight) before ECMO initiation and at 5-180 minutes after the initiation of ECMO [17]. Figure 1 shows a schematic of the experimental design. Saline was administered as a fluid replacement during the experiment, with a total injection volume of 5.0 mL delivered in 1.0 mL increments during blood sampling. After collection, the samples were pipetted into cryovials, which were immediately stored at −80 °C.

Analysis of samples

DEX plasma samples were prepared via protein precipitation and quantified using LC-MS/MS [17]. The method was accurate and precise in the linearity range of 1-100 ng/mL, with intra- and inter-day assay variabilities of <10% for all quality control samples. The analytical validation was treated according to the guidance of the US Food and Drug Administration on bioanalytical method validation [18].

Pharmacokinetics analysis

PK exploration was carried out using two conventional approaches: noncompartmental analysis using moment analysis and compartmental modeling using the NONMEM software version 7.5.1 (ICON Development Solutions, Gaithersburg, Maryland). Different approaches were tested to describe the DEX kinetic profile, while proportional and combined (additive) modeling were tested to determine the residual variability. Once the null model (i.e., a model without factors explaining inter-individual variability) was selected, one documented covariate (ECMO) was tested.

The model was parameterized with the following key PK parameters: volume of distribution (Vd) of the central compartment (V1), clearance (CL), Vd in the peripheral compartment (V2), and intercompartmental CL (Q). Inter-individual variability was modeled using an exponential model as follows:

$\theta_{i}=\theta_{is}*e^{\eta_{i}}$

where $\theta_{i}$ is the estimated individual PK parameter of the $i^{th}$ individual, $\theta_{is}$ the median value of the PK parameter of the population; and $\eta_{i}$ is the inter-individual random effect for the $i^{th}$ individual assumed to be normally distributed with a mean of 0 and a variance of $\omega^{2}$ .

An initial combined proportional and additive residual error model was tested, as expressed in the following equation:

$\Upsilon_{o,ij}=\Upsilon_{p,ij}*(1+\varepsilon_{prop,ij})*\varepsilon_{add,ij}$

where and $\Upsilon_{O,ij}$ are the $j^{th}$ concentrations of the observed and predicted values of the $i^{th}$ individual, where $\varepsilon_{prop,ij}$ is the proportional error, and $\varepsilon_{add,ij}$ is an additional error, with a mean value of 0 and a variance of $\delta^{2}$.

The influence of ECMO was tested using dichotomic covariates of $\theta_{i}$ as follows:

$\theta_{i}=\theta_{pop}&lowast;dic&lowast;e^{\eta_{i}}$

where $\theta_{i}$ is the estimated individual pharmacokinetic parameter for the $i^{th}$ individual, $\theta_{pop}$ is the median value of the PK parameter of the population, and $dic$ is the dichotomic covariate (0 or 1) for the $i^{th}$ individual.

Model evaluation

Goodness-of-fit (GOF) plots were used to evaluate model performance: observed (DV) vs. individual predicted concentrations (IPRED), scatter plots, and residual plots (individual weighted residuals (IWRES) vs. time and predicted concentrations (PRED)). The 95% confidence intervals for the 10th, median, and 90th percentiles of DEX plasma concentration vs. time profiles were plotted to compare observations and model predictions visually.

Statistical analysis

Data were presented in the form of mean ± standard deviation. Continuous variables were compared using repeated-measures one-way analysis of variance and also using Tukey's test. Two-sided P < 0.05 was considered statistically significant. To test potential covariate significance, likelihood ratio tests were performed. A decrease of more than 3.84 in the criteria (P-value = 0.01, χ2 distribution, 1 degree of freedom) was considered significant. The covariates that resulted in a statistically significant improvement (P < 0.05) in the log-likelihood of the model (expressed as an objective function value (OFV)), reduced the residual variability, and/or improved the GOF plots were incorporated to build the final model.

## Results

Changes in DEX concentrations in *in vitro* extracorporeal circuits

**Figure 2:**
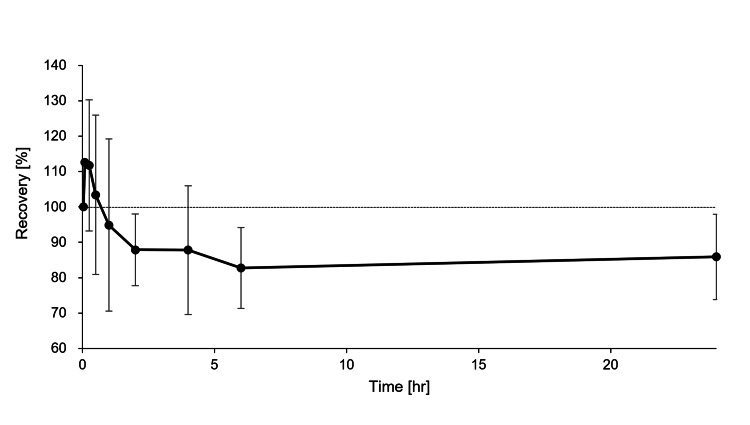
Recovery (%) of DEX measured during the in vitro study. The concentrations are shown for the extracorporeal circuits (n = 3, solid lines). The dotted lines indicate 100% of recovery. At time = 0, DEX (10 µg) was injected as a bolus into the circuit; 0.5 mL of solution was collected at nine time points: two minutes to 24 hours after the initiation. Drug recovery was calculated at each sampling time using the rate for initial concentration at time = two minutes. The values represent the means, and the error bars indicate the standard deviations.

The changes in DEX concentrations during the 24 hours after the injection are shown in Figure 2. The concentration of DEX in the circuit increased immediately after the circuit injection but then decreased, and the recovery rate remained at about 85%.

Change of hemodynamic variables during ECMO on the *in vivo* model

Table 1 shows the changes in each group in terms of the hemodynamic variables PaO_2_ and PaCO_2_, BP, heart rate (HR), pH, and hemoglobin (Hb) concentration. BP and Hb decreased during ECMO in both the ECMO and sham groups. PaO_2_ levels were higher in the ECMO group than in the sham group, although the differences were not statistically significant. Finally, no statistical differences between the groups were recorded with respect to pH or PaCO_2_ levels.

**Table 1:** Hemodynamic variables before and during ECMO Variables are expressed as mean ± standard deviation. PaO_2_: arterial pressure of oxygen; PaCO_2_: arterial pressure of carbon dioxide; BP: blood pressure; HR: heart rate; Hb: hemoglobin

Variable	Group	Pre-ECMO	ECMO 60 min	ECMO 120 min	ECMO 180 min
PaO_2_ (mmHg)	Sham	104±7	103±5	117±14	111±3
	ECMO	104±12	274±37	276±41	268±36
PaCO_2 _(mmHg)	Sham	43±1	38±5	38±2	39±1
	ECMO	41±2	37±3	35±1	35±1
BP (mmHg)	Sham	98±10	78±14	78±11	84±5
	ECMO	104±12	73±5	70±10	69±11
HR (beat/min)	Sham	386±23	369±14	361±7	361±8
	ECMO	403±24	356±14	370±15	353±20
pH	Sham	7.42±0.01	7.37±0.03	7.41±0.02	7.40±0.00
	ECMO	7.41±0.03	7.39±0.01	7.37±0.04	7.36±0.03
Hb (g/dL)	Sham	15.1±1.1	13.2±1.5	12.1±1.2	11.9±1.1
	ECMO	15.3±0.7	10.1±2.4	9.8±1.9	9.3±2.0

Pharmacokinetic analysis

**Figure 3:**
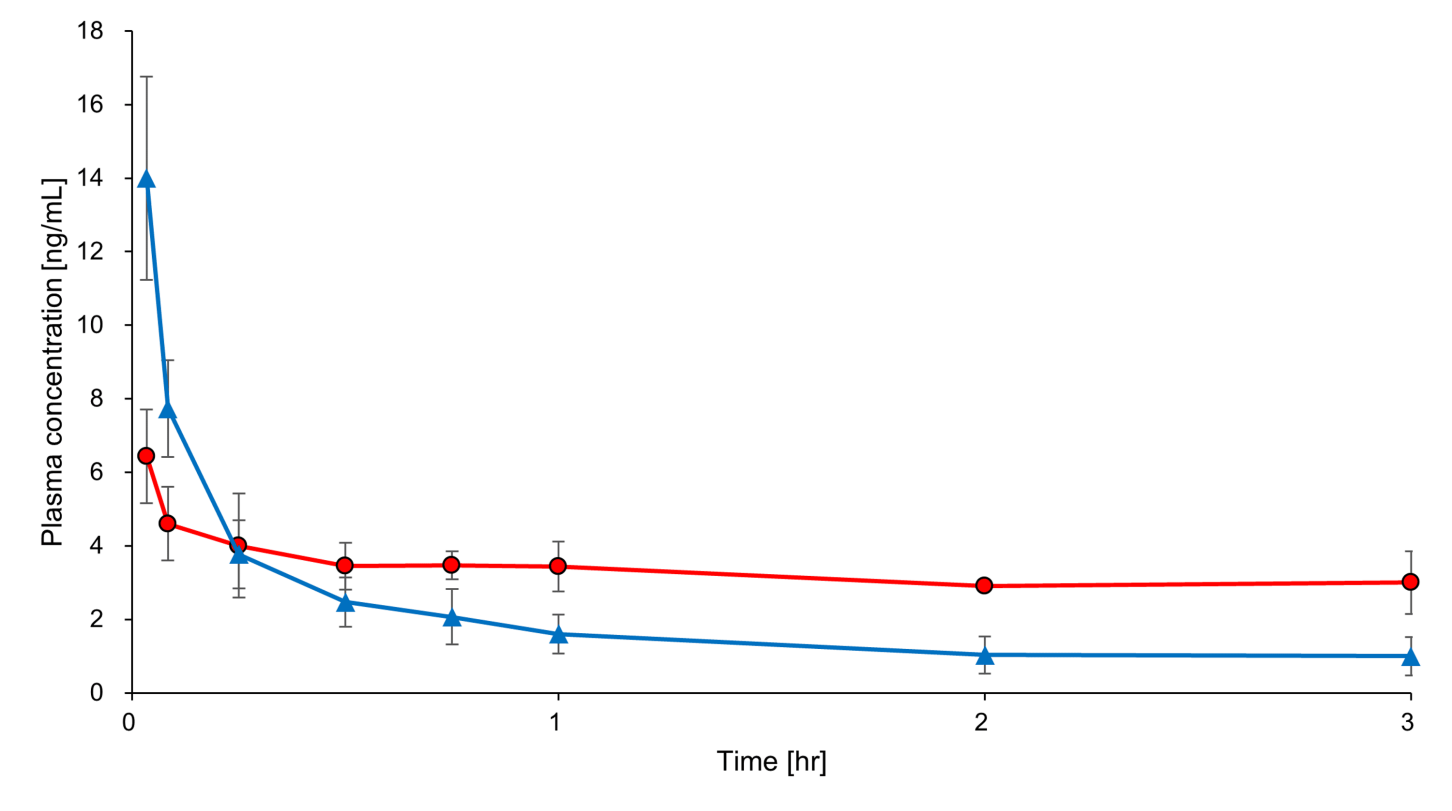
Profiles of plasma concentrations measured during the in vivo study. The blood of each rat was collected, and DEX concentration was measured. ●: ECMO group (n = 3); ▲: sham group (n = 3). Values represent means, and error bars indicate standard deviations. DEX: dexmedetomidine; ECMO: extracorporeal membrane oxygenation

A total of 48 plasma samples were collected from six rats and measured for DEX concentrations. DEX concentration profiles obtained from the ECMO and sham groups are presented in Figure 3.

Using the noncompartmental approach, it was found that among the mean individual parameters determined for the ECMO and the sham groups, the area under the curve (AUC_0→180 minutes_), mean residence time (MRT), t_1/2_, and steady-state volume of distribution (Vd_ss_) all increased in the ECMO group compared with the sham group, whereas the total clearance (CL_tot_) decreased (Table 2).

**Table 2:** PK parameters estimated using a noncompartmental analysis AUC: area under the curve; MRT: mean residence time; CL_tot_: total clearance; Vd: volume of distribution

Parameter	Dexmedetomidine	
	ECMO	Sham
AUC_0→180 min_(ng/mL・min)	485.2 ± 54.8	367.0 ± 95.2
MRT (min)	1703.4 ± 1438.8	165.8 ± 90.2
t_1/2 _(min)	1186.6 ± 995.7	141.4 ± 61.5
CL_tot _(L/h)	0.20 ± 0.21	1.59 ± 0.92
Vd_ss_(L/kg)	5.63 ± 0.43	5.62 ± 0.76

The best-fitting model for describing the PK profile of DEX was a two-compartment model with a proportional error. The PK parameter estimates, interindividual variability, and residue errors for the final model are summarized in Table 3.

**Table 3:** Parameters estimated by the population PK model ECMO: extracorporeal membrane oxygenation; PK: pharmacokinetics; CV: coefficient of variation; CL: clearance; V1: volume of distribution in the central compartment; V2: volume of distribution in the peripheral compartment; Q: intercompartmental clearance

Parameter (unit)	Final model	
	Mean	% RSE
Typical value		
θ_CL__・__Sham _(L/hr)	1.15	54.0
θ_CL__・__ECMO _(L/hr)	0.34	201.8
θ_V1 _(L)	0.93	22.8
θ_V2 _(L)	2.33	20.8
θ_Q_ (L/hr)	5.32	15.4
ECMO	0.29	262.9
Interindividual variability, CV%		
ω_CL_	19.0	71.8
ω_V1_	2.6	65.6
Residual variability		
Additive, ng/mL	0.15	53.2
Proportional, %	12.9	74.1

**Figure 4:**
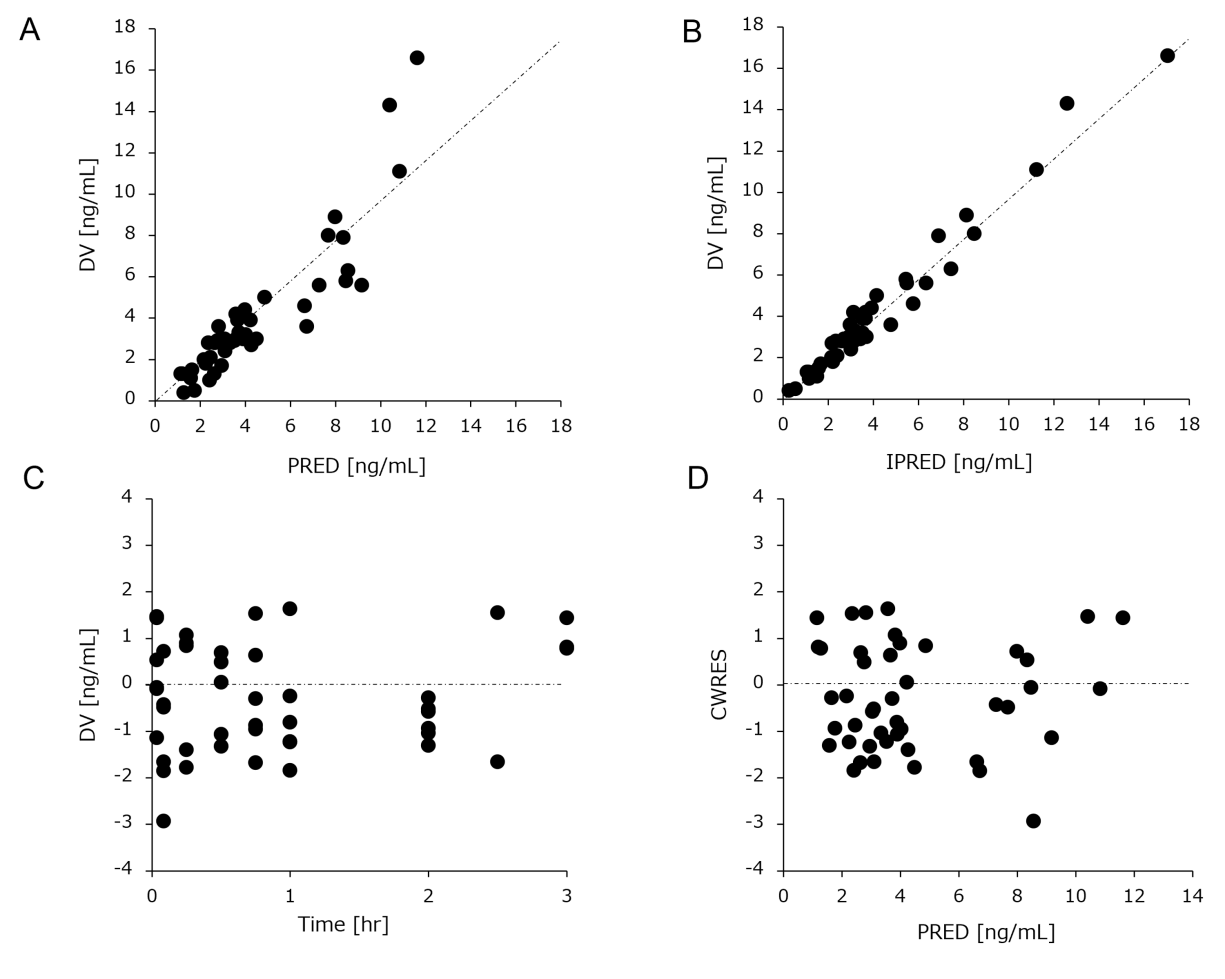
Goodness-of-fit plots for the final pharmacokinetics model. The plots show the (A) population predictions and (B) individual predictions vs. observations, (C) time, and (D) population predictions vs. conditional weighted residuals. The dotted lines in the top panels (A and B) represent the line of identity, and the dotted lines in the lower panels (C and D) represent zero lines.

The typical value of CL was 1.15 (L/hour) in the sham group and 0.34 (L/hour) in the ECMO group. The typical values of V1 and V2 were 0.93 L and 2.33 L, respectively. The typical value of Q was 5.32 (L/hour). The estimated inter-individual variability (expressed as coefficient of variation, CV%) was 19 for CL and 2.6 for V1. The ECMO significantly influenced the CL of DEX, so that CL was 75% lower in the presence of ECMO compared with the absence of ECMO. Subsequent to a visual check of prespecified covariates, the effect of the covariates on PK parameters was then assessed using ECMO. As shown in Figure 4, the basic GOF plots revealed that the final model was acceptable because the predicted population and individual concentrations closely aligned with the observed concentrations.

**Figure 5:**
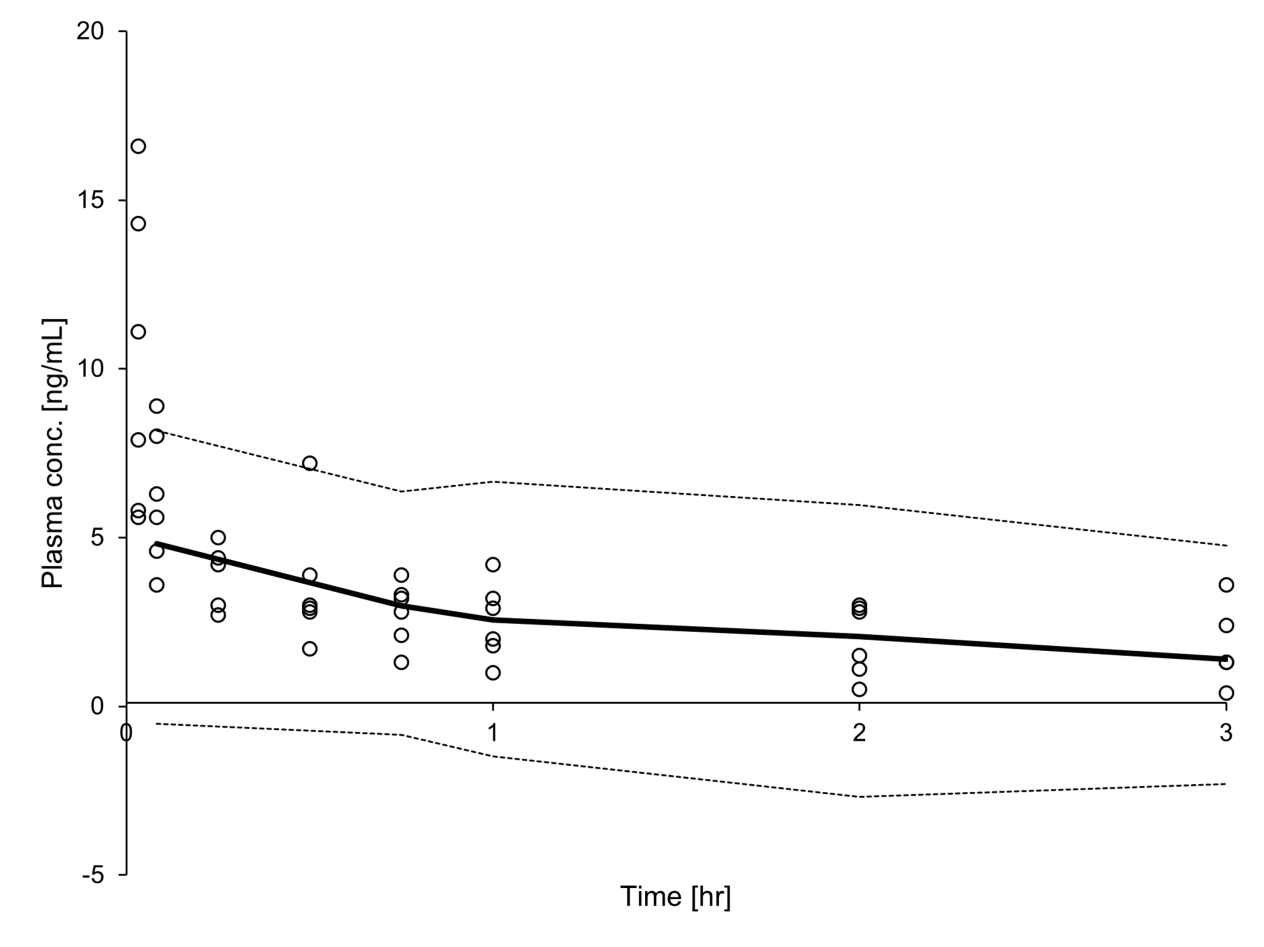
Visual predictive check of the final population pharmacokinetic model for DEX during ECMO. Open cycles indicate observed DEX concentrations; the solid line represents the median; the lower and upper dashed lines represent the 10th and 90th percentiles, respectively, of the simulated data. DEX: dexmedetomidine; ECMO: extracorporeal membrane oxygenation

Most conditional weighted residual values were evenly distributed in a random manner around the line of unity (± 2 standard deviations of the mean), which indicated the suitability of the error model. The proportion of observations between the 10th and 90th simulated percentiles was >90%, which was deemed acceptable (Figure 5).

## Discussion

In this study, we investigated an *in vitro* extracorporeal circuit, which did not incorporate a membrane oxygenator; instead, the following were used: a reservoir bottle, a centrifugal pump with PVC tubing, a sampling port, and injection ports. DEX was then injected into the circuit. As a result, the expected recovery (%) of DEX decreased after injection. DEX is a hydrophobic drug that is highly protein-bound (94%). The adsorption of albumin on PVC tubing might increase the sequestration of drugs that have a high rate of protein binding [19]. It is a well-known principle that proteins, in general, are adsorbed by artificial surfaces, depending on the surface charge and the proteins involved [20,21]. In this study, DEX adsorption in the circuit was transient but only slightly, which is in line with our previous results. The results obtained from *in vitro* models do not suggest an effect of circuits on DEX concentrations.

For *in vivo* experiments, the human adult ECMO device was reduced to 1/50-1/100, and a rat model was selected [13]. In this study, a two-compartment model was selected, as used previously in the population PK analysis of DEX in humans [6]. The changes in PK/pharmacodynamics parameters are necessary for ECMO therapy [13]. According to Shekar et al., an increase in protein-unbound drugs and Vd reduces plasma levels in neonates [22]. Hemodilution of cardiac output and drug adsorption are considered factors that increase Vd. Some reports show that the Vd of lipophilic drugs increases in patients [17].

DEX is a highly protein-bound drug. In plasma, 94% of DEX is bound to albumin and α1-glycoprotein [13]. For DEX, both prolonged [23] and shortened [24] elimination half-times have been reported in patients with hypoalbuminemia. The total CL of DEX is affected by the concentration of plasma albumin. In our *in vivo* studies, ECMO decreased in total CL (0.34 vs. 1.15 L/hour) using the NONMEM approach and a decrease in total CL (0.20 vs. 1.59 L/hour) according to noncompartmental analysis. DEX is metabolized mainly by the liver, and a hepatic extraction ratio of 0.7 was obtained in the previous study [5]. DEX has been reported to be dependent on rates of hepatic blood flow. In our study, BP and HR were lower in the ECMO group than in the sham group. DEX CL is associated with changes in liver blood flow, which may correlate with reduced cardiac output [25]. Fluctuations in vitals might also be affected by DEX administration. Similar fluctuations in vitals were found to occur in a previous study, which used the same ECMO rat model as in the present work, suggesting that these fluctuations are due to the ECMO [13]. Therefore, if a patient is suspected of having reduced ECMO-related CL, a slight overexposure to DEX can be expected. These results suggest that ECMO may affect the efficacy or produce side effects associated with DEX.

The experiments conducted in the present study had limitations. First, we did not use a membrane oxygenator in the *in vitro* experiment. Because DEX is mainly adsorbed by the tubes in the ECMO circuit, it is necessary to investigate the change of concentration in the circuit, which incorporates a membrane oxygenator. Second, this was a pilot study with a small sample size. Future studies will allow us to elucidate the sequential mechanisms of the pathological condition by evaluating the histopathological severity of disorders. Finally, we evaluated the PK during short-term ECMO. Further studies are required to assess the systemic inflammatory response and organ damage during long-term ECMO and after weaning off ECMO.

## Conclusions

In the present study, we obtained preliminary data on DEX PK obtained using an ECMO model. Population PK models were also developed for study purposes. The ECMO model represented decreased CL compared with the non-ECMO group. Larger studies are needed to characterize the disposition of DEX during ECMO. If a patient is suspected of having reduced ECMO-related clearance, overexposure to DEX can be expected. These results suggest that ECMO may affect the efficacy or produce side effects associated with DEX.
